# Meloxicam Alleviates Sepsis-Induced Lung Injury by Inhibiting Pyroptosis Through CBP/TXNIP/p38 Signaling Pathway

**DOI:** 10.3390/ph19060929

**Published:** 2026-06-12

**Authors:** Lixia Cheng, Qian Li, Yuting Liu, Jiahao Liu, Jianqi Zhao, Linfeng Wang, Meiling Liu, Xiaowen Bi, Chunhong Huang

**Affiliations:** 1 Department of Biochemistry and Molecular Biology, School of Basic Medical Sciences, Jiangxi Medical College, Nanchang University, Nanchang 330006, China; chenglixia99@163.com (L.C.); 15736745922@163.com (Q.L.); liujiahao4035@163.com (J.L.); zhaojq2001@163.com (J.Z.); wanglinfeng2002@163.com (L.W.); 13517180571@163.com (M.L.); 2 Department of Medical Genetics and Cell Biology, School of Basic Medical Sciences, Jiangxi Medical College, Nanchang University, Nanchang 330006, China; liuyuting5114@163.com

**Keywords:** sepsis-induced lung injury, meloxicam, pyroptosis, NLRP3 inflammasome, CBP/TXNIP/p38 signaling pathway

## Abstract

**Background:** Macrophage pyroptosis contributes substantially to sepsis-induced lung injury, yet effective therapeutic strategies remain limited. This study aimed to determine the protective effects of meloxicam, a non-steroidal anti-inflammatory drug, and the underlying mechanisms in this context. **Methods:** *In vivo*, CLP mice were treated with meloxicam (20 mg/kg). *In vitro*, LPS-primed macrophages were stimulated with ATP or nigericin in the presence or absence of meloxicam. Levels of pyroptosis-associated proteins (cleaved Caspase-1, mature IL-1β, GSDMD-NT), NLRP3 inflammasome assembly, and the CBP/TXNIP/p38 signaling axis were assessed by Western blot. Mitochondrial membrane potential (ΔΨm) and intracellular ROS were measured. Overexpression of COX-2, TXNIP, and CBP was also performed. **Results:** Meloxicam significantly improved survival, reduced lung injury, and suppressed pyroptosis-associated proteins in CLP mice. In vitro, meloxicam dose-dependently enhanced macrophage viability and reduced LDH, IL-1β, and IL-18 release. The protective effects of meloxicam were mediated by inhibition of NLRP3 inflammasome priming and assembly, disruption of NLRP3-ASC-pro-Caspase-1 complex formation, and suppression of ASC oligomerization. Meloxicam also inhibited the CBP/TXNIP/p38 axis, an effect reversed by TXNIP or CBP overexpression. Furthermore, meloxicam restored ΔΨm and reduced ROS accumulation; these effects were abrogated by the ROS inducer imiquimod. Importantly, the anti-pyroptotic effects of meloxicam were independent of COX-2 inhibition. **Conclusions:** These findings expand the pharmacological profile of meloxicam and support its repurposing as a therapeutic agent for sepsis-associated lung injury.

## 1. Introduction

Sepsis refers to a life-threatening organ dysfunction resulting from a dysregulated host response to infection, with inflammatory imbalance being a hallmark throughout its progression [1,2]. Excessive and uncontrolled inflammation not only leads to direct tissue damage but also precipitates multiple organ dysfunction syndrome (MODS), which significantly contributes to sepsis-related mortality [3]. Among affected organs, the lung is particularly susceptible. Sepsis-induced lung injury, a common and severe complication, often presents as acute lung injury (ALI) or its more extreme form, acute respiratory distress syndrome (ARDS), both of which significantly worsen patient prognosis [4]. Critically, inter-organ crosstalk contributes significantly to the pathogenesis and progression of sepsis-related ALI/ARDS, underscoring the complexity of systemic inflammatory responses in sepsis [5,6]. Therefore, targeted therapeutic strategies require a thorough elucidation of the molecular mechanisms underlying sepsis-induced lung injury, which is essential for improving clinical outcomes.

Emerging evidence implicates pyroptosis, mediated by inflammatory Caspases, as a pivotal contributor to sepsis-induced tissue damage [7]. Pyroptosis is a form of programmed cell death, hallmarked by the rupture of the plasma membrane and the extracellular release of intracellular pro-inflammatory cytokines, including IL-1β and IL-18, which further amplify the inflammatory response [8]. In sepsis, macrophage pyroptosis—a process in these key innate immune effector cells-promotes the release of excessive pro-inflammatory mediators, infiltration of inflammatory cells, and disruption of alveolar architecture, which in turn exacerbates lung injury [8,9,10].

The classical pyroptosis pathway, commonly known as the Caspase-1-dependent pathway, is primarily triggered by activation of the nucleotide-binding oligomerization domain (NOD)-like receptor family pyrin domain-containing 3 (NLRP3) inflammasome [11]. In sepsis, the NLRP3 inflammasome, assembled from NLRP3, the apoptosis-associated speck-like protein containing a Caspase recruitment domain (CARD) (ASC), and pro-Caspase-1, is excessively assembled and exhibits hyperactivation, leading to macrophage pyroptosis [12]. The activation process of the NLRP3 inflammasome involves a two-step process. The priming stage is triggered by damage-associated molecular patterns (DAMPs) and pathogen-associated molecular patterns (PAMPs), which upregulate the expression levels of NLRP3, pro-Caspase-1 and pro-IL-1β via activating the nuclear factor kappa-light-chain-enhancer of activated B cells (NF-κB) signaling. The assembly stage involves a second signal, mediated by factors such as potassium efflux, mitochondrial dysfunction, and lysosomal damage, which promotes the assembly of NLRP3, ASC, and caspase-1 into an active NLRP3 inflammasome, resulting in Caspase-1 activation. Activated Caspase-1 can cleave pro-IL-1β and pro-IL-18 into their mature forms (IL-1β and IL-18), and also process gasdermin D (GSDMD) into the N-terminal fragment of gasdermin D (GSDMD-NT), initiating pyroptosis and driving inflammation [13,14,15]. Importantly, inhibition of NLRP3 inflammasome activation has been shown to suppress macrophage pyroptosis and mitigate ALI [13,16,17], suggesting that targeting this pathway represents a promising therapeutic strategy [18].

Emerging evidence indicates that the transcriptional co-activator and histone acetyltransferase CREB-binding protein (CBP) is markedly upregulated and hyper-activated across a spectrum of pathologies, including malignancy, chronic inflammation, and cardiovascular disorders, thereby critically driving disease progression [19,20]. Pharmacological targeting of the CBP bromodomain has been demonstrated to alleviate lethal sepsis through downregulating HMGB1 expression and antagonizing its pro-inflammatory functions [21]. Meanwhile, thioredoxin-interacting protein (TXNIP), a stress response protein, further amplifies pyroptosis under oxidative stress via multiple mechanisms, including NLRP3 inflammasome activation and modulation of the mitogen-activated protein kinase (MAPK) signaling pathway [22,23]. The MAPK signaling cascade is a central mediator of sepsis-associated inflammation. Activation of pattern recognition receptors such as Toll-like receptors (TLRs) and DAMP receptors triggers a phosphorylation cascade involving RAF, MEK, ERK, and MAPK family members, which subsequently activate transcription factors including AP-1 and NF-κB, driving the production and release of pro-inflammatory mediators [24]. As a central component of the MAPK pathway, p38 exhibits increased phosphorylation levels that are closely linked to diverse inflammatory responses [25] and has been demonstrated to potentiate NLRP3 inflammasome formation and subsequent pyroptosis [26]. Collectively, these observations position CBP-mediated inflammatory cascades and TXNIP-driven inflammasome activation as two mechanistically distinct yet potentially synergistic checkpoints whose simultaneous modulation may offer a tractable therapeutic strategy in sepsis.

Meloxicam [27,28], a nonsteroidal anti-inflammatory drug (NSAID), exhibits potent anti-inflammatory, analgesic, and antipyretic properties, with a high selectivity for inhibiting the inducible cyclooxygenase-2 (COX-2) isoform over the constitutive cyclooxygenase-1 (COX-1), thereby minimizing gastrointestinal and renal adverse effects [29]. Clinically, meloxicam is widely utilized for the management of chronic inflammatory disorders, such as rheumatoid arthritis and osteoarthritis [30]. Beyond its classical COX-2 inhibition, meloxicam has demonstrated anti-tumor activities by suppressing COX-2 overexpression in various cancers, including colorectal cancer [31], hepatoma [32], osteosarcoma [33], and esophageal squamous cell carcinoma [34], thereby inhibiting cancer cell proliferation, invasion, and migration [35,36,37]. Recent studies further reveal that meloxicam exerts effects independent of COX-2 inhibition [38,39,40]. For example, meloxicam promotes AXL receptor tyrosine kinase (AXL) degradation by disrupting the Hsp90-CDC37 complex, thereby suppressing melanoma metastasis and recurrence [41], and suppresses hepatocellular carcinoma progression by activating AMPK and promoting mTOR phosphorylation [42]. Despite these advances, the pharmacological potential of meloxicam in sepsis, particularly in modulating macrophage pyroptosis and lung injury, remains poorly understood.

Therefore, in this study, we demonstrate that meloxicam confers substantial protection against sepsis-induced lung injury by suppressing macrophage pyroptosis and attenuating the associated inflammatory response. Mechanistically, meloxicam inhibits NLRP3 inflammasome activation and the downstream pyroptotic cascade via suppression of the CBP/TXNIP/p38 signaling axis, an effect mediated by the restoration of mitochondrial membrane potential and the attenuation of reactive oxygen species (ROS) accumulation. To the best of our knowledge, this is the first study to demonstrate that meloxicam exerts its anti-pyroptotic effects through a COX-2-independent mechanism. These findings expand the pharmacological repertoire of meloxicam beyond its conventional anti-inflammatory actions and support its potential repositioning as a therapeutic candidate for sepsis-associated acute lung injury, a condition for which targeted therapies remain limited.

## 2. Results

### 2.1. Meloxicam Reduces Lung Injury and Pyroptosis in Septic Mice

**Figure 1:**
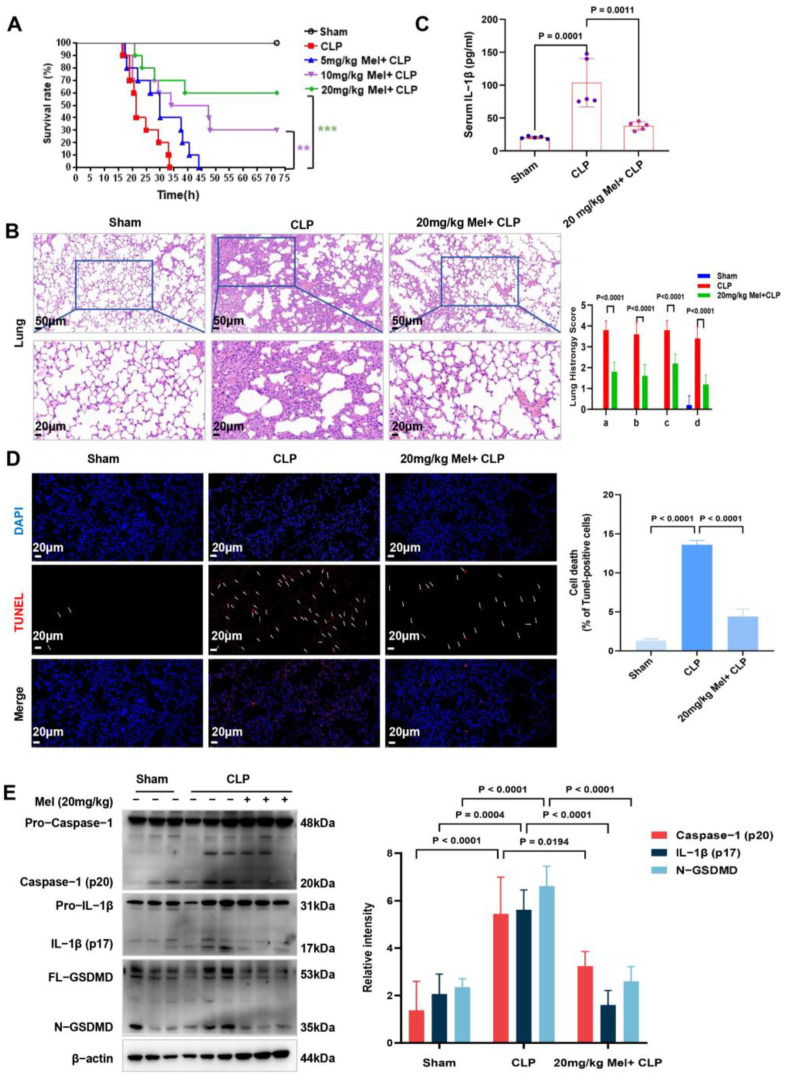
Meloxicam attenuates lung injury and suppresses pyroptosis in septic mice. (**A**) Male BALB/c mice were intraperitoneally administered meloxicam (5, 10 or 20 mg/kg) or vehicle 2 h before undergoing CLP surgery, and survival was continuously monitored for 72 h. Sham surgery mice served as controls. *n* = 10 mice/group. ** *p* < 0.01, *** *p* < 0.001. (**B**–**E**) Male BALB/c mice were given intraperitoneal injections of meloxicam (20 mg/kg) or vehicle 2 h prior to undergoing CLP or sham operation. Lung tissue and serum were collected 18 h post-procedure. Lung tissue sections were stained with H&E and examined by light microscopy. Scale bars: 20 µm and 50 µm. *n* = 5 mice/group. The following parameters were evaluated: (a) alveolar septal thickness, (b) interstitial edema, (c) infiltration of inflammatory cells, (d) alveolar congestion/collapse (**B**). Serum IL-1β levels were quantified by ELISA. *n* = 5 mice/group (**C**). TUNEL and DAPI co-stained lung sections were analyzed by fluorescence microscopy. Scale bars: 20 µm. *n* = 5 mice/group (**D**). Expression of pyroptosis-associated proteins in lung tissue was assessed by Western blotting. *n* = 3 mice/group (**E**). Data are presented as mean ± SD.

To evaluate how meloxicam protects against lung injury caused by sepsis, we injected mice with meloxicam (5, 10 or 20 mg/kg) prior to CLP treatment. As shown in Figure 1A, meloxicam dose-dependently improved the survival rates of septic mice, with the 20 mg/kg dose exhibiting the most significant effect. Thus, this optimal dose was selected for the subsequent experiments. Histological analysis (H&E staining) revealed significant lung injury in the CLP group, characterized by alveolar collapse, overdistension, septal disruption, and inflammatory cell infiltration, all of which were significantly alleviated by meloxicam treatment (Figure 1B). Consistently, ELISA results showed that meloxicam treatment significantly decreased serum IL-1β levels in septic mice (Figure 1C). Immunofluorescence analysis further demonstrated extensive DNA fragmentation in lung tissues after CLP, indicative of pyroptosis—a form of inflammatory programmed cell death implicated in sepsis pathogenesis [43]. Meloxicam administration markedly reduced this DNA fragmentation (Figure 1D). To directly evaluate pyroptosis, we analyzed key pyroptosis-associated proteins. Western blotting analysis revealed elevated levels of activated Caspase-1 (p20), mature IL-1β, and GSDMD-NT in lung tissues from CLP-challenged mice, and meloxicam treatment reduced these levels (Figure 1E). These results indicate that meloxicam effectively attenuated sepsis-induced lung injury and inhibited pyroptosis in pulmonary tissue.

### 2.2. Meloxicam Inhibits Pyroptosis of Macrophages

**Figure 2:**
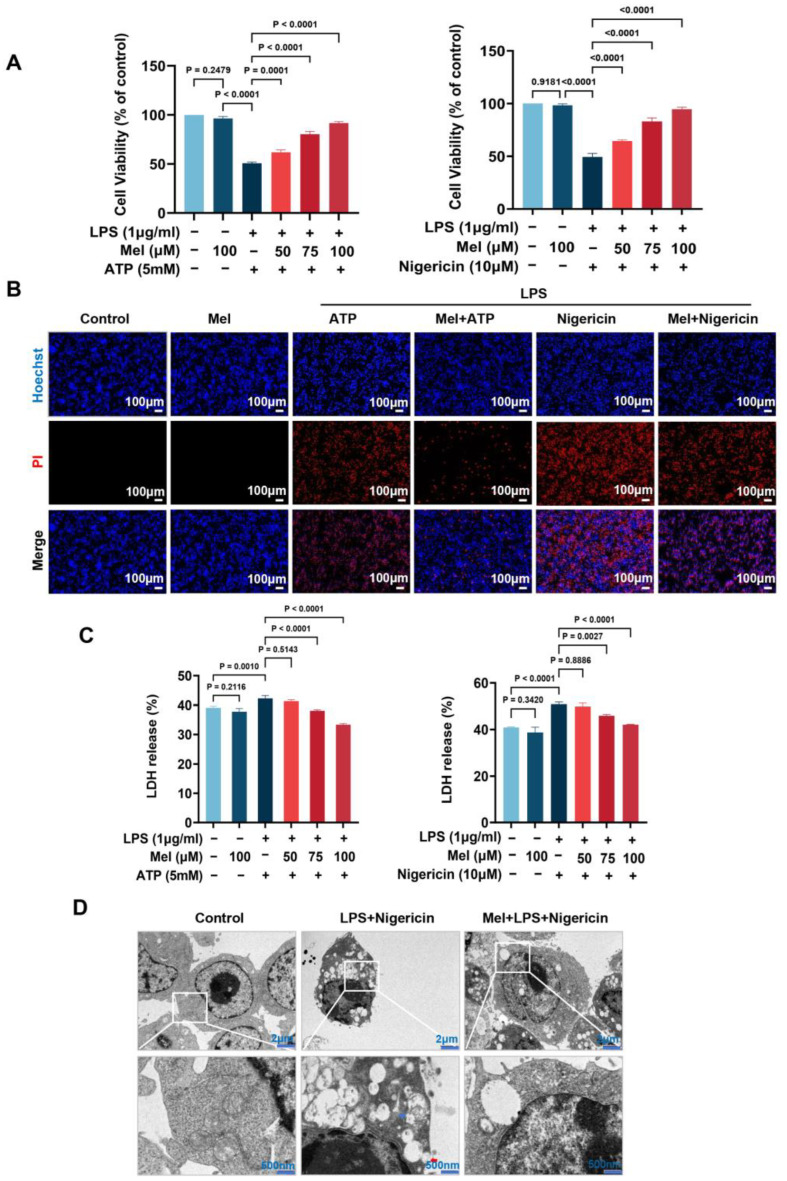

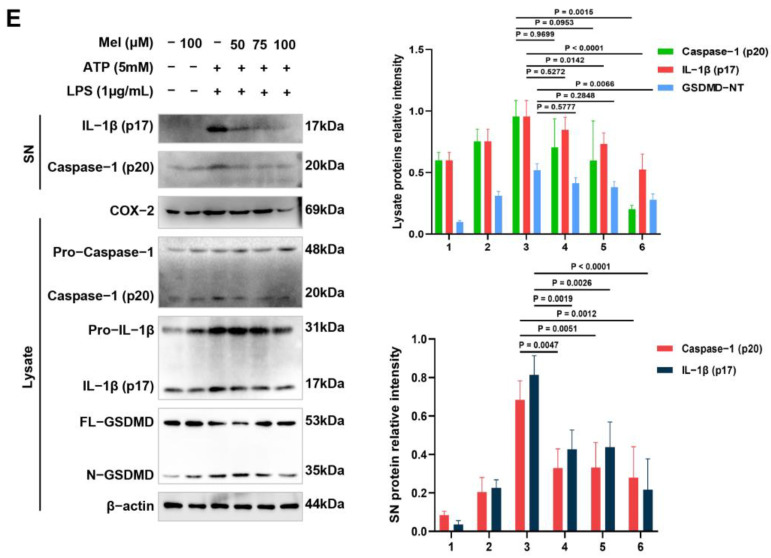
Meloxicam inhibits macrophage pyroptosis in a dose-dependent manner. (**A**) RAW264.7 cells were pretreated with meloxicam (50, 75, or 100 µM) for 2 h, prior to being stimulated with LPS (1 µg/mL) for 18 h plus Nigericin (10 µM, 1 h) or ATP (5 µM, 1 h). Cell viability was measured using the CCK-8 assay (*n* = 5). (**B**) Following pretreatment with meloxicam (100 µM, 2 h), RAW264.7 cells were stimulated with LPS (1 µg/mL, 18 h) and then treated with Nigericin (10 µM, 1 h) or ATP (5 µM, 1 h). Cell pyroptosis was assessed by Hoechst/PI staining (*n* = 3). Scale bars: 100 µm. (**C**) RAW264.7 cells underwent the same treatment protocol as in (**A**), and LDH release was measured to assess cell membrane integrity (*n* = 5). (**D**) RAW264.7 cells underwent the same treatment protocol as in (**B**), and ultrastructural changes were examined using TEM (*n* = 3). Scale bars: 2 µm and 500 nm. (**E**) After pretreatment with meloxicam (50, 75, or 100 µM) for 2 h, RAW264.7 cells were incubated with LPS (1 µg/mL, 18 h) and then treated with ATP (5 µM, 1 h), and Western blotting analysis was conducted to assess the expression of pyroptosis-associated proteins (*n* = 3). Data are presented as mean ± SD.

To investigate the effect of meloxicam on macrophage pyroptosis, we conducted in vitro experiments using RAW264.7 cells and PMA-differentiated THP-1 cells. Meloxicam significantly improved cell viability that had been reduced by LPS combined with ATP or Nigericin stimulation (Figure 2A and Figure S1A). Hoechst/PI staining further validated that meloxicam attenuated LPS + ATP/Nigericin-induced pyroptotic cell death in RAW264.7 cells (Figure 2B). The hallmarks of pyroptosis include membrane pore formation, cellular swelling, membrane breakdown, and the secretion of pro-inflammatory cytokines and other cellular contents [44]. We measured LDH, IL-1β and IL-18 levels in the cell culture supernatants to assess this process. An LDH release assay revealed that meloxicam dose-dependently suppressed the elevated LDH levels induced by LPS + ATP/Nigericin in both RAW264.7 cells and PMA-differentiated THP-1 cells (Figure 2C and Figure S1B). Similarly, ELISA analysis showed that meloxicam suppressed the increase in IL-1β and IL-18 induced by LPS + Nigericin in PMA-induced THP-1 cells in a dose-dependent manner (Figure S1C). Furthermore, TEM images revealed that meloxicam attenuated pyroptotic ultrastructural changes in LPS + Nigericin-stimulated RAW264.7 cells, including membrane pore formation, cytoplasmic dissolution, vacuolization, and nuclear membrane rupture, and also alleviated mitochondrial damage (Figure 2D). Western blotting analysis confirmed that LPS + ATP stimulation in RAW264.7 cells and LPS + Nigericin treatment in PMA-induced THP-1 cells markedly increased the levels of activated Caspase-1 (p20), mature IL-1β (in both supernatants and lysates), and GSDMD-N (in lysates). These increases were significantly suppressed by meloxicam treatment (Figure 2E and Figure S1D). Collectively, these results indicate that meloxicam effectively inhibited macrophage pyroptosis in a dose-dependent manner.

### 2.3. Meloxicam Inhibits the Activation of NLRP3 Inflammasomes

**Figure 3:**
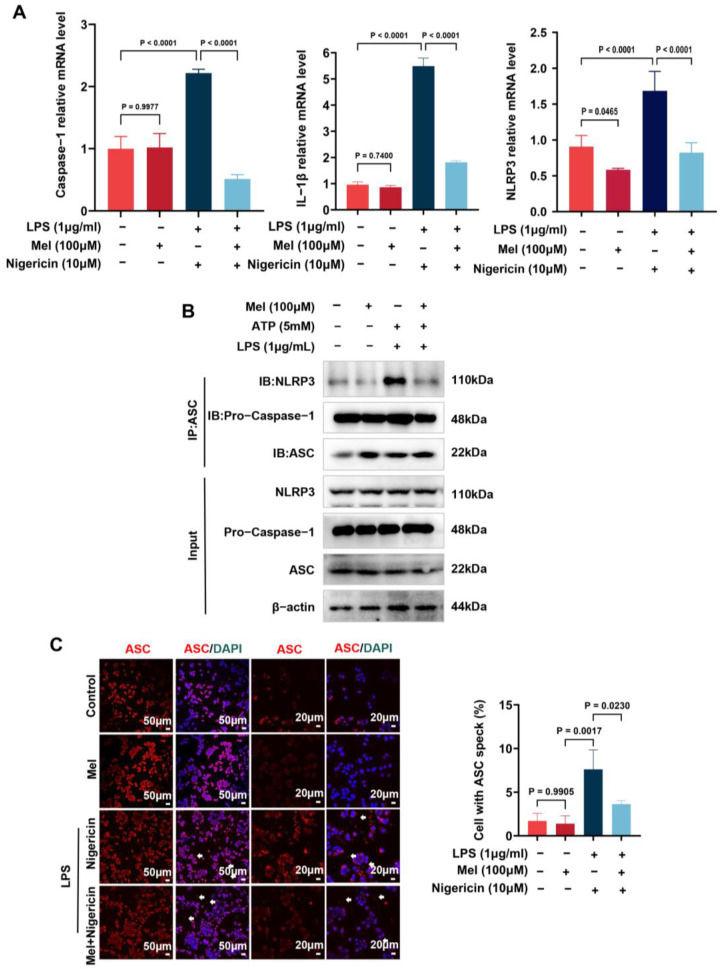
Meloxicam inhibits NLRP3 inflammasome activation. (**A**–**C**) After incubation with meloxicam (100 µM) for 2 h, RAW264.7 cells were exposed to LPS (1 µg/mL) for 18 h and then stimulated with Nigericin (10 µM) for 1 h or ATP (5 µM) for 1 h. Real-time quantitative PCR was performed to measure mRNA levels of *Caspase-1*, *IL-1β*, and *NLRP3* (*n* = 3) (**A**). Co-immunoprecipitation was conducted to detect the interactions among NLRP3, ASC, and pro-Caspase-1 (**B**). ASC specks were detected using laser confocal microscopy (*n* = 3). Scale bars: 20 µm and 50 µm (**C**). Data are presented as mean ± SD.

To determine whether meloxicam targets the priming and assembly stages of NLRP3 inflammasome activation in macrophages, we performed the following experiments. RT-PCR analysis showed that stimulation with LPS plus Nigericin significantly increased the mRNA levels of *Caspase-1, IL-1β*, and *NLRP3* in RAW264.7 cells, while this upregulation was significantly attenuated by meloxicam (Figure 3A). These results demonstrate that meloxicam could inhibit the priming phase of NLRP3 inflammasome activation. NLRP3 inflammasome assembly relies on the oligomerization of NLRP3, ASC, and pro-Caspase-1 [13]. Co-immunoprecipitation assays demonstrated that the NLRP3-ASC-pro-Caspase-1 interaction was enhanced in both LPS + ATP-stimulated RAW264.7 cells and LPS + Nigericin-stimulated THP-1 cells that had been induced with PMA, and this interaction was disrupted by meloxicam (Figure 3B and Figure S2A). During inflammasome assembly, activated ASC oligomerizes into cytoplasmic specks, forming ASC specks [45], which serve as a hallmark of NLRP3 inflammasome activation. In RAW264.7 cells, laser confocal microscopy revealed that meloxicam substantially reduced LPS + Nigericin-induced ASC speck formation (Figure 3C). Collectively, these results indicate that meloxicam effectively inhibits both the priming and assembly phases of NLRP3 inflammasome activation.

### 2.4. Meloxicam Suppresses NLRP3 Inflammasome Activation Through Regulating the CBP/TXNIP/p38 Signaling Pathway

**Figure 4:**
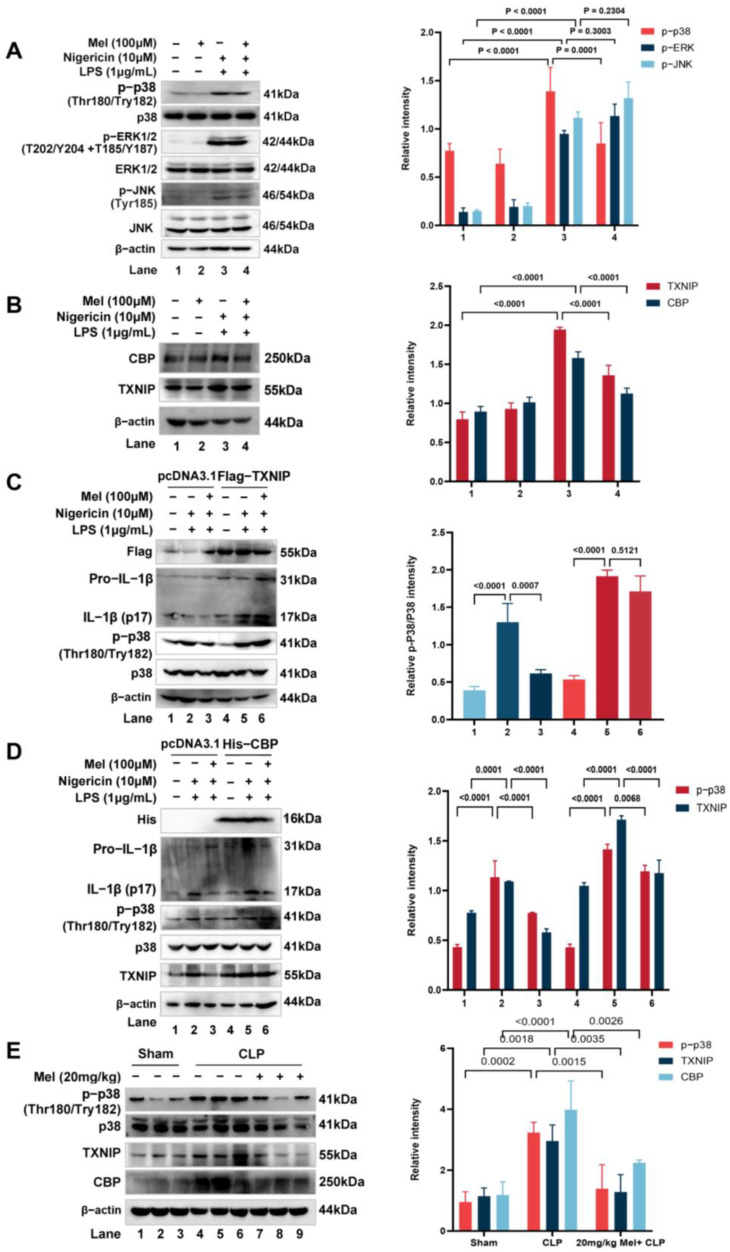
Meloxicam inhibits the activation of NLRP3 inflammasomes through downregulating the CBP/TXNIP/p38 signaling pathway. (**A**,**B**) After pretreatment with meloxicam (100 µM, 2 h), RAW264.7 cells were stimulated with LPS (1 µg/mL, 18 h) plus Nigericin (10 µM, 1 h). Western blotting analysis was performed to detect total and phosphorylated forms of p38, ERK, and JNK, as well as TXNIP and CBP levels (*n* = 3). (**C**) Following transfection with *Flag-TXNIP* or *pcDNA3.1*, RAW264.7 cells were pretreated with meloxicam (100 µM, 2 h), then treated with LPS (1 µg/mL, 18 h) plus Nigericin (10 µM, 1 h) or ATP (5 µM, 1 h). Western blotting analysis was used to analyze the protein levels of p38, p-p38, pro-IL-1β and IL-1β (p17) (*n* = 3). (**D**) After transfection with His-CBP (3WDY: bromodomain plasmid fragment) or pcDNA3.1, RAW264.7 cells were pretreated with meloxicam (100 µM, 2 h), then stimulated with LPS (1 µg/mL, 18 h) plus Nigericin (10 µM, 1 h) or ATP (5 µM, 1 h). Western blotting analysis was performed to analyze the protein levels of p38, p-p38, pro-IL-1β, IL-1β (p17) and TXNIP (*n* = 3). (**E**) Male BALB/c mice were intraperitoneally injected with meloxicam (20 mg/kg, 2 h) prior to CLP, with sham-operated mice as controls. Lung tissues were collected 18 h post-surgery, and Western blotting analysis was performed to detect p38, p-p38, TXNIP and CBP levels (*n* = 3). Data are presented as mean ± SD.

To elucidate how meloxicam inhibits NLRP3 inflammasome activation and pyroptosis, we assessed the phosphorylation status of key MAPK pathway proteins (p38, ERK, and JNK) by Western blotting. As shown in Figure 4A and Figure S3A, meloxicam selectively suppressed the phosphorylation level of p38 in both LPS plus Nigericin-induced RAW264.7 cells and PMA-differentiated THP-1 cells stimulated with LPS plus ATP, without significantly affecting phosphorylated ERK or JNK levels, which suggests that meloxicam may inhibit the activation of NLRP3 inflammasome via blocking the phosphorylation of p38. Further Western blotting results indicated that the elevated TXNIP levels were significantly reduced through meloxicam treatment (Figure 4B and Figure S3B). Given that TXNIP regulates p38 phosphorylation [23], we overexpressed Flag-TXNIP in RAW264.7 cells. TXNIP overexpression increased p-p38 and IL-1β levels, partially reversing the inhibitory effects of meloxicam on these proteins (Figure 4C). We next examined CBP, which we previously found elevated in inflammatory models [21,46]. CBP was similarly upregulated in pyroptosis models, and meloxicam was able to downregulate these elevated CBP levels (Figure 4B and Figure S3B). Given that p300, a CBP homolog, regulates the transcription of the *TXNIP* [47], we examined whether meloxicam inhibits TXNIP expression via CBP. His-CBP (3WDY: bromodomain plasmid fragment) was transfected into RAW264.7 cells. As shown in Figure 4D, CBP overexpression significantly increased TXNIP levels and partially restored p-p38 and IL-1β levels that were suppressed by meloxicam. Consistent with cellular findings, Western blotting analysis of lung tissues from septic mice showed that meloxicam suppressed CBP, TXNIP, and phosphorylated p38 levels in vivo (Figure 4E). In summary, these findings demonstrate that meloxicam suppressed pyroptosis and activation of the NLRP3 inflammasome by modulating the CBP/TXNIP/p38 signaling pathway.

### 2.5. Meloxicam Inhibits CBP/TXNIP/p38 Signaling Pathway by Reducing ROS Accumulation

**Figure 5:**
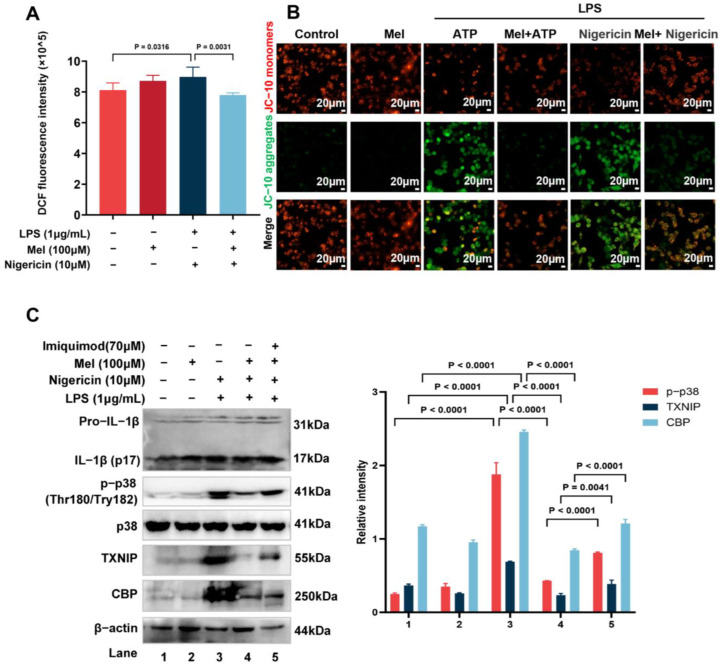
Meloxicam suppresses the CBP/TXNIP/p38 pathway through ROS regulation. (**A**) RAW264.7 cells received meloxicam (100 µM) pretreatment for 2 h before being stimulated with LPS (1 µg/mL, 18 h) plus Nigericin (10 µM, 1 h) (*n* = 5). The accumulation of intracellular ROS was measured. (**B**) RAW264.7 cells were incubated with meloxicam (100 µM) for 2 h and exposed to LPS (1 µg/mL, 18 h) plus Nigericin (10 µM, 1 h) or ATP (5 µM, 1 h). Cells were stained with JC-10 and images were captured using a fluorescence microscope (*n* = 3). Scale bar: 20 µm. (**C**) RAW264.7 cells were incubated with imiquimod (70 µM, 2 h) prior to meloxicam (100 µM) treatment for 2 h, then stimulated with LPS (1 µg/mL, 18 h) plus Nigericin (10 µM, 1 h). Western blotting was performed to assess the protein levels of p38, p-p38, pro-IL-1β, IL-1β (p17), TXNIP and CBP (*n* = 3). Data are presented as mean ± SD.

ROS regulate CBP levels [48]. To investigate whether the effect of meloxicam on the CBP/TXNIP/p38 pathway is ROS-dependent, we measured intracellular ROS levels. Meloxicam significantly reduced LPS + Nigericin-induced ROS accumulation in both RAW264.7 and PMA-differentiated THP-1 cells (Figure 5A and Figure S4A). Given the contribution of ΔΨm disruption to ROS accumulation [49], JC-10 staining was employed to examine ΔΨm. The result revealed that meloxicam restored ΔΨm in LPS + ATP/Nigericin-stimulated RAW264.7 cells (Figure 5B). This finding validated our previous observation that meloxicam alleviated mitochondrial damage in LPS + Nigericin-stimulated RAW264.7 cells (Figure 2D). To further confirm that meloxicam inhibits ROS production, RAW264.7 cells were pretreated with imiquimod, an ROS inducer, before LPS + Nigericin stimulation. Imiquimod reversed the inhibitory effects of meloxicam on CBP, TXNIP and p-p38 protein levels (Figure 5C). These findings indicate that meloxicam inhibited the CBP/TXNIP/p38 signaling pathway by alleviating mitochondrial membrane damage and reducing intracellular ROS accumulation.

### 2.6. Meloxicam Inhibits the CBP/TXNIP/p38 Signaling Pathway via a COX-2-Independent Mechanism

**Figure 6:**
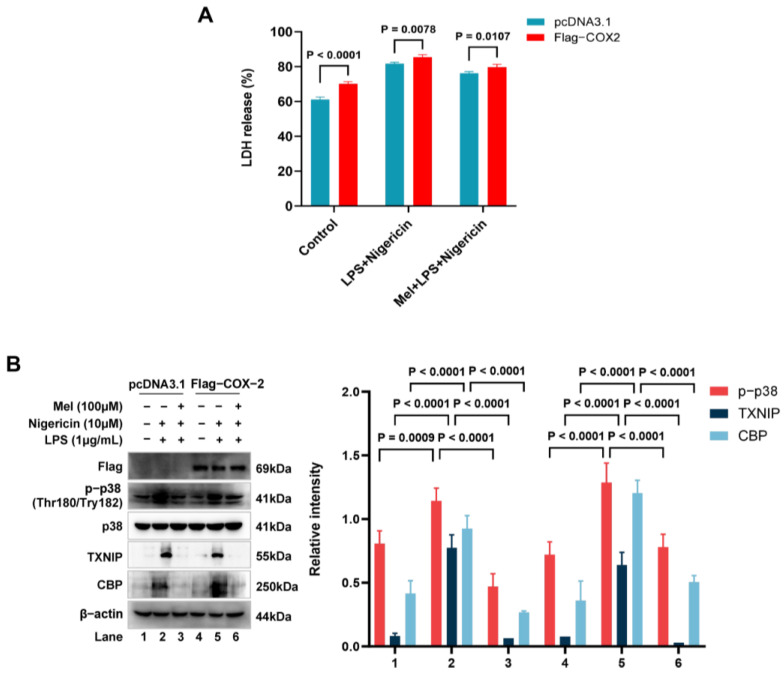
The inhibition of the CBP/TXNIP/p38 signaling pathway by meloxicam via a COX-2-independent mechanism. (**A**,**B**) After transfection with *pcDNA3.1* or *Flag-COX-2*, RAW264.7 cells were incubated with meloxicam (100 µM) for 2 h, followed by LPS (1 µg/mL, 18 h) plus Nigericin (10 µM, 1 h). The levels of extracellular LDH were determined (*n* = 5) (**A**). The protein levels of p38, p-p38, TXNIP and CBP were analyzed by Western blotting (*n* = 3) (**B**). Data are presented as mean ± SD.

To determine whether meloxicam’s suppression of NLRP3 inflammasome activation and pyroptosis requires COX-2 inhibition, we overexpressed Flag-COX-2 in LPS + Nigericin-stimulated RAW264.7 cells. Notably, COX-2 overexpression failed to reverse the inhibitory effect of meloxicam on LDH release (Figure 6A). Additionally, Western blotting analysis revealed that Flag-COX-2 overexpression partially restored CBP levels and p38 phosphorylation, which were suppressed by meloxicam, while TXNIP levels remained unaffected (Figure 6B). Thus, these results indicate that meloxicam regulates the CBP/TXNIP/p38 axis independently of COX-2 inhibition.

## 3. Discussion

Sepsis-induced lung injury remains a major clinical challenge, largely driven by dysregulated inflammatory responses and excessive cell death [50]. A key pathological mechanism in sepsis is pyroptosis, a form of programmed inflammatory cell death that largely depends on activation of the NLRP3 inflammasome [8,9]. Our study establishes meloxicam, a conventional NSAID [29], as a novel therapeutic agent that significantly improves survival and alleviates sepsis-induced lung damage in mice. Crucially, we demonstrate that this protection stems from meloxicam’s unique ability to inhibit macrophage pyroptosis through suppression of NLRP3 inflammasome activation, extending beyond its canonical COX-2 inhibitory function. This positions meloxicam as a dual-mechanism agent capable of simultaneously addressing inflammation and programmed cell death in sepsis pathophysiology. To distinguish pyroptosis-specific inhibition from generalized anti-inflammatory effects, we specifically quantified hallmarks of pyroptosis, including GSDMD-NT cleavage, ASC speck formation, and LDH release—none of which are markers of classical apoptosis or general inflammation suppression. The consistent suppression of these pyroptosis-specific endpoints across multiple stimuli (ATP, nigericin) and cell models supports the conclusion that meloxicam exerts genuine anti-pyroptotic activity.

A central finding of our work is the identification of the CBP/TXNIP/p38 axis as a critical regulatory pathway modulated by meloxicam to inhibit NLRP3 inflammasome-mediated pyroptosis. The transcriptional co-activator CBP, known for its histone acetyltransferase activity, is increasingly recognized as a pivotal epigenetic regulator of inflammatory gene expression [51]. Our data suggest that meloxicam suppresses CBP levels, thereby downregulating TXNIP, a redox-sensitive protein that directly interacts with NLRP3 to trigger inflammasome assembly [52,53]. This is mechanistically supported by the established role of p300/CBP in transcriptionally activating TXNIP expression via carbohydrate response element-binding protein (ChREBP)-mediated histone acetylation at the TXNIP promoter [46]. As a close homolog of p300, CBP likely regulates TXNIP transcription through a similar mechanism. The functional relevance of this relationship was confirmed by our CBP overexpression experiments, which restored TXNIP levels and partially reversed meloxicam’s inhibitory effects on p-p38 and IL-1β. This insight reveals an intricate crosstalk between epigenetic modification and redox signaling in controlling inflammasome activation, emphasizing the importance of transcriptional regulation in sepsis pathogenesis. Furthermore, the restoration of mitochondrial function and reduction in ROS by meloxicam underscore the interconnectedness of metabolic and epigenetic regulation, as ROS levels influence CBP stability and activity. This multilayered regulation highlights potential therapeutic leverage points to disrupt the feed-forward loop of oxidative stress and inflammation. It should be noted that our study does not provide evidence for direct physical interaction between meloxicam and NLRP3 inflammasome components. Rather, the data support an indirect mechanism whereby meloxicam suppresses upstream regulators (CBP, TXNIP, ROS) to prevent inflammasome assembly. Whether meloxicam can directly bind NLRP3 or ASC remains an open question that warrants future investigation using biophysical approaches such as surface plasmon resonance or thermal shift assays.

The MAPK pathway, activated downstream of TLRs and DAMP receptors through the RAF–MEK–ERK–MAPK phosphorylation cascade, is a well-established driver of inflammatory mediator production in sepsis [24]. Under oxidative stress conditions, TXNIP translocates from the nucleus to the cytoplasm, where it binds to thioredoxin 1 (TRX1) and subsequently activates the p38 signaling pathway [23]. p38 is a crucial mediator of inflammatory responses, with its phosphorylation closely associated with inflammation [50,54], and has been implicated in regulating NLRP3 inflammasome activity [26]. The selective inhibition of p38 phosphorylation by meloxicam, without affecting ERK or JNK pathways, further refines our understanding of the signaling specificity involved in inflammasome activation. The selectivity of meloxicam for p38 over ERK and JNK may be explained by the upstream TXNIP-dependent mechanism. Under oxidative stress, TXNIP preferentially activates the p38 pathway through its interaction with apoptosis signal-regulating kinase 1 (ASK1), a known upstream activator of p38 but not ERK or JNK under these conditions [23,52]. By reducing TXNIP levels, meloxicam selectively disrupts this ASK1–p38 axis without broadly suppressing MAPK signaling, which is consistent with the observed specificity. Given p38’s established role in mediating inflammatory cytokine production and cell death, its modulation by meloxicam via the CBP/TXNIP axis positions this pathway as a critical node for therapeutic intervention. Notably, TXNIP overexpression reverses meloxicam’s inhibitory effects on p-p38 and IL-1β maturation, confirming the functional relevance of this signaling cascade in regulating pyroptosis. These findings suggest that targeting upstream regulators of p38 activation, such as CBP and TXNIP, may provide more selective and effective strategies to mitigate sepsis-induced inflammation compared to direct kinase inhibition, which often suffers from off-target effects. Furthermore, while TXNIP overexpression experiments supported the functional role of TXNIP in the CBP/TXNIP/p38 axis, complementary loss-of-function studies using shRNA-mediated TXNIP knockdown or CRISPR-based gene editing were not performed. Such experiments would provide stronger causal evidence and represent an important direction for future work.

Notably, meloxicam’s upstream regulation of this cascade reveals an unexpected role in mitochondrial homeostasis. Mitochondria, as the primary source of intracellular ROS [55], can generate excessive ROS when ΔΨm is disrupted [49,56]. In sepsis, mitochondrial dysfunction and ROS accumulation are key contributors to pyroptosis and tissue injury [57]. Notably, previous studies have demonstrated that ROS can increase cellular CBP levels by reducing CBP degradation without necessarily altering its transcription [48]. Our data demonstrate that meloxicam preserves cristae architecture and ΔΨm integrity. The functional significance of this mitochondrial protection was confirmed when ROS induction with imiquimod abolished meloxicam’s effects on CBP levels, indicating that mitochondrial stabilization initiates the entire protective cascade. This positions meloxicam among rare therapeutics capable of concurrently targeting organelle dysfunction and inflammatory cell death, a strategy potentially applicable to other ROS-driven pathologies like ischemia–reperfusion injury. It should be noted that imiquimod, in addition to inducing intracellular ROS, may also activate TLR7/8-mediated immune signaling pathways [23]. Therefore, the reversal effects observed with imiquimod pretreatment should be interpreted with the caveat that some contribution from TLR7/8 activation cannot be entirely excluded. Future studies using alternative ROS inducers (e.g., H_2_O_2_ or rotenone) would help confirm the ROS-specific nature of these observations.

Importantly, our data indicate that meloxicam’s anti-pyroptotic effects occur independently of COX-2 inhibition. While COX-2 overexpression failed to reverse pyroptosis inhibition, we observed partial restoration of CBP and p-p38 expression, suggesting complex crosstalk between prostaglandin and TXNIP pathways. Although COX-2 overexpression partially restored CBP and p-p38 levels, TXNIP remained unaffected, suggesting that prostaglandin signaling may modulate certain upstream nodes of the pathway without being essential for the overall anti-pyroptotic effect. The contribution of specific prostaglandin receptors (e.g., EP2/EP4) to NLRP3 inflammasome regulation warrants further investigation, as PGE2-EP receptor signaling has been reported to both promote and suppress inflammasome activation depending on cellular context [57]. This echoes emerging evidence that NSAIDs possess “off-target” effects on kinase networks and transcriptional regulators [38]. Clinically, this duality may confer unique advantages: COX-2 inhibition mitigates early hyperinflammation [58], while CBP/TXNIP/p38 suppression prevents later pyroptotic tissue damage. Such temporal synergy could explain meloxicam’s superior efficacy in our model compared to other selective COX-2 inhibitors. This challenges the traditional view that NSAID efficacy is predominantly mediated through COX-2 blockade and invites further investigation into alternative molecular targets. Understanding these COX-2-independent mechanisms may facilitate the development of novel NSAID derivatives or combination therapies that maximize anti-inflammatory benefits while minimizing side effects associated with COX inhibition.

Regarding the clinical relevance of the doses employed, the in vivo dose of 20 mg/kg in mice corresponds to an estimated human equivalent dose of approximately 1.6 mg/kg based on body surface area normalization, which falls within the clinically approved dosing range for meloxicam (7.5–15 mg/day for adults) [28,29]. The in vitro concentrations used (50–100 µM) are higher than typical steady-state plasma concentrations achieved clinically; however, such concentrations are widely employed in macrophage-based mechanistic studies to achieve sufficient intracellular drug exposure and are consistent with published in vitro mechanistic studies of meloxicam in inflammatory cell models [27]. Future studies using lower, clinically relevant concentrations in primary human macrophages will be important to confirm translational applicability.

These findings illuminate new therapeutic horizons for sepsis management. The CBP/TXNIP/p38 axis represents a druggable target for pyroptosis-driven pathologies beyond sepsis, including ARDS, pancreatitis, and gouty inflammation [49]. Moreover, the identification of a COX-2-independent anti-pyroptotic mechanism opens new avenues for the rational design of next-generation NSAID derivatives that selectively target the CBP bromodomain or TXNIP–NLRP3 interaction, potentially offering superior efficacy with reduced gastrointestinal and cardiovascular side effects. The combination of meloxicam with mitochondrial-targeted antioxidants may also represent a promising synergistic strategy worthy of future investigation. Meloxicam’s established safety profile and blood–brain barrier permeability further support its repurposing potential [59]. Nevertheless, several limitations of the present study must be acknowledged. First, although the murine CLP model recapitulates many features of human sepsis, substantial interspecies differences exist in inflammasome activation and immune responses [2]. Validation in primary human alveolar macrophages and clinical sepsis tissue samples is therefore warranted. Second, the survival analysis was limited to a 72-h observation window, which may not capture late-phase sepsis events such as immunoparalysis or secondary infection-related mortality [3]. Future studies employing extended observation periods are needed to assess long-term outcomes. Third, only male BALB/c mice were used in this study. Given that sex-dependent differences in NLRP3 inflammasome activation and inflammatory responses may influence therapeutic outcomes [9], inclusion of female animals in future studies is necessary. Fourth, the in vitro concentrations of meloxicam used in this study (up to 100 µM) exceed typical clinical plasma levels. Although such concentrations are routinely used in macrophage-based mechanistic studies and are lower than those reported in previous investigations without apparent cytotoxicity [27], their clinical translatability requires further validation using primary human macrophages at lower concentrations. Fifth, although this study demonstrated the acute protective effects of short-term meloxicam administration, its long-term safety profile in septic conditions was not evaluated. As a preferential COX-2 inhibitor, meloxicam still retains some inhibitory activity against COX-1, which may contribute to gastrointestinal, renal, and cardiovascular adverse effects, particularly at high doses [28,29]. Future studies should therefore include comprehensive safety assessments, particularly regarding long-term tolerability, before clinical translation. Moreover, while this study focused on pyroptosis, other forms of programmed cell death—including apoptosis, ferroptosis, and necroptosis—may also contribute to septic lung injury [9]. Whether meloxicam modulates these pathways in addition to pyroptosis warrants investigation in future studies. Additionally, although meloxicam restored mitochondrial membrane potential and ultrastructural integrity, direct measurements of mitochondrial oxygen consumption rate and ATP production were not performed. These functional assessments will be essential in future studies to fully elucidate meloxicam’s effects on mitochondrial bioenergetics. Finally, although our study employed multiple complementary approaches to demonstrate NLRP3 inflammasome inhibition, genetic validation using NLRP3-knockout mice or specific pharmacological inhibitors such as MCC950 would provide stronger mechanistic confirmation and represents an important future direction.

## 4. Materials and Methods

### 4.1. Reagents, Antibodies and Plasmids

LPS (from *Escherichia coli* O111:B4) and ATP were purchased from Sigma-Aldrich (St. Louis, MO, USA). Nigericin (S6653) was obtained from Selleck Chemicals (Houston, TX, USA). Imiquimod and meloxicam were purchased from Target Mol (Boston, MA, USA).

Antibodies against Caspase-1, COX-2, NLRP3, ASC, and p-ERK1/2 (T202/Y204+T185/Y187) were purchased from ABclonal (Wuhan, China). Antibodies targeting p38, ERK, JNK, p-JNK (Tyr185), DYKDDDDK (Flag tag), β-actin and His-tag were purchased from Proteintech (Rosemont, IL, USA). Antibodies targeting IL-1β, TXNIP, and CBP were obtained from Cell Signaling Technology (Beverly, MA, USA). Antibodies against GSDMD were purchased from Affinity Biosciences (Shanghai, China). Antibodies against p-p38 (Thr180/Tyr182) were sourced from ZEN-BIOSCIENCE (Chengdu, Sichuan, China).

*His-CBP* (3WDY: bromodomain plasmid fragment) was acquired from DeTaiBio (Nanjing, China). *Flag-TXNIP* was obtained from the Public Protein/Plasmid Library (Nanjing, China). *Flag-COX-2* was obtained from Sino Biological (Beijing, China). All plasmids were confirmed via DNA sequencing (Genscript, Piscataway, NJ, USA) and then purified via the Endofree Plasmid Preparation Kit (Qiagen, Hilden, Germany).

### 4.2. Animal Model of Cecal Ligation and Puncture (CLP)-Induced Sepsis

Male BALB/c mice (six weeks old, 20 to 22 g) were purchased from GemPharmatech Co., Ltd. (Nanjing, Jiangsu, China; Permit Number: SCXK (Su) 2023–0009). The experiments adhered to the provisions of the Chinese Guidelines for the Care and Use of Laboratory Animals. The animal experiments involved in this study were approved by the Committee on Animal Experimental Ethical Inspection of Nanchang University [Number of experimental facility certification: SYXK (Gan) 2021–0004]. The Approval Number is NCULAE–20220624026.

Mice were randomly divided into five groups: sham surgery, CLP model, and CLP treated with meloxicam at doses of 5, 10, or 20 mg/kg. Two hours prior to surgery, mice received intraperitoneal injections of either vehicle or meloxicam at the specified doses. Anesthesia was induced by intraperitoneal injection of sodium pentobarbital (30 mg/kg). Once anesthetized, mice were fixed on a surgical board, and the abdominal area was disinfected. A 1-cm midline incision was made to expose the cecum, which was isolated and ligated 1.5 cm from the tip with a 3-0 silk suture. An 18-gauge needle was used to puncture the cecum twice between the ligation site and the tip. This resulted in the extrusion of a small amount (droplets) of feces. The incision was sutured closed, and 1 mL of prewarmed normal saline was administered intraperitoneally to compensate for fluid loss. Sham-operated mice underwent the same procedure without cecal ligation or puncture. Mortality was monitored continuously for 72 h.

To specifically assess the effect of meloxicam on lung injury in septic mice, animals were divided into three groups: sham surgery, CLP model, and CLP treated with meloxicam (20 mg/kg), following the same treatment protocol. At 18 h post-surgery, serum and lung tissues were collected for hematoxylin and eosin (H&E) staining, enzyme-linked immunosorbent assay (ELISA), Western blotting, and terminal deoxynucleotidyl transferase dUTP nick end labeling (TUNEL) immunofluorescence staining.

### 4.3. Hematoxylin and Eosin Staining

After isolation, mouse lung tissues were fixed in 4% paraformaldehyde, embedded in paraffin, and cut into serial sections. These sections were then stained with H&E for histopathological examination. Blinded assessment was carried out at the same magnification for all samples.

Lung injury was evaluated by light microscopic analysis of four parameters: alveolar septal thickness, interstitial edema, infiltration of inflammatory cells, and alveolar congestion/collapse. Each parameter was graded into four categories based on the percentage of affected area: 0 = normal; 1 = ≤25%; 2 = 25–50%; 3 = 50–75%; 4 = ≥75%. The mean score of the four parameters was calculated and used to represent the overall lung injury score for each mouse.

### 4.4. Cell Culture and Transfection

RAW264.7 cells (murine macrophage-like) and THP-1 cells (human acute monocytic leukemia) were purchased from the Cell Bank of the Chinese Academy of Sciences (Shanghai, China). RAW264.7 cells were cultured in DMEM (Bio-Channel, Nanjing, China) supplemented with 10% (*v*/*v*) fetal bovine serum (FBS; ExCell Bio, Shanghai, China) and an antibiotic–antimycotic mixture (100 U/mL penicillin, 0.1 mg/mL streptomycin, and 0.25 µg/mL amphotericin B mixture; Biosharp, Beijing, China). THP-1 cells were maintained in RPMI 1640 (ViVaCell, Shanghai, China) supplemented with 15% (*v*/*v*) FBS and the same antibiotic–antimycotic mixture (Biosharp). Both cell lines were maintained at 37 °C in a humidified 5% CO_2_ atmosphere. THP-1 cells were differentiated using phorbol 12-myristate 13-acetate (PMA, 100 ng/mL, 12 h).

Cells were transfected with *His-CBP* (3WDY: bromodomain plasmid fragment), *Flag-TXNIP*, *Flag-COX-2*, or the control vector *pcDNA3.1* using X-tremeGENE HP DNA Transfection Reagent (Sigma-Aldrich), according to the manufacturer’s instructions. The total DNA amount in each transfection was equalized by the addition of an empty control plasmid.

### 4.5. Cell Viability and Cytokine Assays

Cell viability was assessed using a Cell Counting Kit-8 (CCK-8; Abbkine, Wuhan, China), following the manufacturer’s instructions. Serum samples from each mouse group and cell culture supernatants were collected. According to the manufacturer’s protocol, ELISA kits (Elabscience, Wuhan, China) were used to quantify IL-1β and IL-18 concentrations.

### 4.6. Cell Death Detection

Cell death was assessed using two methods: (1) Propidium Iodide (PI) Staining, cell culture supernatants were collected and incubated with PI and Hoechst working solution (Elabscience) at room temperature for 10 min, followed by fluorescence microscopy imaging; (2) Lactate Dehydrogenase (LDH) Release, 120 μL of cell supernatant was mixed with 60 μL of LDH assay reagent (Beyotime, Shanghai, China) in a 96-well plate. After a 30-min dark incubation, the absorbance was read at 490 nm.

### 4.7. Reverse Transcription Polymerase Chain Reaction (RT-PCR)

Total RNA was extracted from cells using an RNA extraction kit (Monad Biotech, Wuhan, China), and cDNA was synthesized with a reverse transcription kit (Monad Biotech). Quantitative PCR was subsequently performed using SYBR Green premix (Monad Biotech) according to the manufacturer’s instructions.

The following primers were used for RT-PCR:

Mouse-*GSDMD*-Forward GCAAATTCAACGGCACAGTCAAG

Mouse-*GSDMD*-Reverse TCGCTCCTGGAAGATGGTGATG

Mouse-*NLRP3*-Forward CCAGACCTCCAAGACCACTACG

Mouse-*NLRP3*-Reverse CAGAGAAGAGATGCTCCTCAATGC

Mouse-*IL-1β*-Forward GCAACTGTTCCTGAACTCAACT

Mouse-*IL-1β*-Reverse ATCTTTTGGGGTCCGTCAACT

Mouse-*Caspase-1*-Forward ACAAGGCACGGGACCTATG

Mouse-*Caspase-1*-Reverse TCCCAGTCAGTCCTGGAAATG

### 4.8. Western Blotting Analysis

Cells were lysed in RIPA lysis buffer (Solarbio, Beijing, China) supplemented with protease and phosphatase inhibitor cocktail (Target Mol). Total protein concentration was quantified with the BCA Protein Assay Kit (Beyotime, Shanghai, China). Equivalent protein amounts from each sample were resolved by SDS-PAGE and subsequently electrotransferred onto PVDF membranes (Sigma-Aldrich). The membranes were blocked with 5% skim milk powder for 1 h at room temperature, followed by overnight incubation at 4 °C with the primary antibody. After washing, the membranes were incubated with HRP-conjugated secondary antibody for 1 h at ambient temperature. Chemiluminescent signals from Western blot membranes were captured using the Tanon 5200 chemiluminescence imaging system. Quantitative densitometric analysis of the protein bands was subsequently performed using the accompanying Tanon Image analysis software (Tanon, Shanghai, China).

### 4.9. Co-Immunoprecipitation (Co-IP) Assays

Equal amounts of proteins (500 μg/sample) were subjected to immunoprecipitation with the indicated antibodies at 4 °C overnight, then incubated for 3 h with protein A/G plus-agarose beads (Santa Cruz Biotechnology, Dallas, TX, USA). Following four washes with lysis buffer, the immunoprecipitated complexes were boiled in SDS sample buffer. Target proteins in the precipitated pellets were analyzed by Western blotting.

### 4.10. Confocal Laser-Scanning Microscopy

Cells were fixed for 30 min in 4% paraformaldehyde and then permeabilized for 5 min using 0.1% Triton X-100. Cells were then blocked with 5% BSA for 1 h and incubated overnight at 4 °C with the primary antibody targeting ASC. The cells were then incubated with a fluorescent dye-conjugated secondary antibody for 1 h in the dark. The nuclei were stained with 4′,6-diamidino-2-phenylindole (DAPI) and visualized using a confocal laser scanning microscope.

### 4.11. Transmission Electron Microscopy (TEM)

Cells were harvested, fixed in 2.5% glutaraldehyde for 2 h at room temperature, and post-fixed with 1% osmium tetroxide. Samples were dehydrated through an ethanol series, embedded in epoxy resin, and polymerized at 60 °C for 48 h. Ultrathin sections were cut using an ultramicrotome, stained with uranyl acetate and lead citrate, and observed using a transmission electron microscope.

### 4.12. Measurement of ROS Generation

Intracellular ROS levels were determined using an ROS kit (Beyotime, Shanghai, China) per the manufacturer’s instructions, and cells were finally viewed under a fluorescence microscope.

### 4.13. Mitochondrial Membrane Potential (ΔΨm) Measurement

Assessment of ΔΨm was performed using JC-10 (4A Biotech, Suzhou, China). Cells, after treatment, were incubated with JC-10 working solution for 30 min at room temperature in the dark. After three washes with PBS, fluorescence images were taken using a fluorescence microscope.

### 4.14. Statistical Analysis

Data are presented as mean ± standard deviation (SD). GraphPad Prism 9.0 software (GraphPad Software, San Diego, CA, USA) was used for data analysis and graphing. The normality of data distribution was assessed using the Shapiro–Wilk test, and homogeneity of variance was evaluated using the Brown–Forsythe test or Bartlett’s test. The data met parametric test assumptions. Accordingly, an unpaired Student’s *t*–test was used to compare differences between two groups. One–way analysis of variance (ANOVA) followed by Tukey’s post hoc test was used for comparisons among multiple groups.

## 5. Conclusions

**Figure 7:**
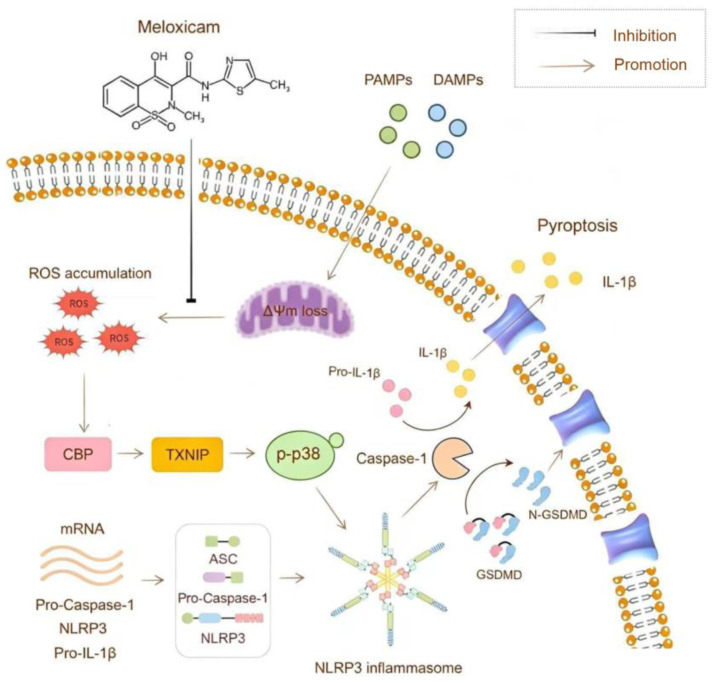
Schematic diagram illustrating the proposed mechanism by which meloxicam inhibits NLRP3 inflammasome-mediated pyroptosis. In damaged lung tissue, meloxicam ameliorates mitochondrial dysfunction and attenuates ROS production, thereby suppressing the activation of the CBP/TXNIP/p38 signaling axis. This inhibition subsequently attenuates NLRP3 inflammasome-mediated pyroptosis, ultimately alleviating inflammation and tissue injury.

In summary, this study elucidates a novel mechanism by which meloxicam attenuates sepsis-induced lung injury through the suppression of the CBP/TXNIP/p38 signaling axis, mediated in part by the restoration of mitochondrial function and reduction in ROS (Figure 7). These insights enrich our comprehension of the molecular interplay between epigenetic regulation, redox signaling, and inflammasome activation in sepsis and provide a mechanistic foundation for repurposing meloxicam as a therapeutic agent for sepsis-associated acute lung injury.

## Data Availability

The original contributions presented in this study are included in the article/Supplementary Material. Further inquiries can be directed to the corresponding authors.

## Supplementary Materials

The following supporting information can be downloaded at: https://www.mdpi.com/article/10.3390/ph19060929/s1, Figure S1: Meloxicam exerts a dose-dependent inhibitory effect on macrophage pyroptosis. (A) After being pretreated with meloxicam (50, 75, or 100 µM) for 2 h, PMA-induced THP-1 cells were incubated with LPS (1 µg/mL, 18 h), followed by Nigericin (10 µM, 1 h). The CCK-8 assay was used to evaluate cell viability (*n* = 5). (B) PMA-induced THP-1 cells were pretreated with meloxicam (50, 75, or 100 µM) for 2 h, prior to LPS (1 µg/mL, 18 h) plus Nigericin (10 µM, 1 h) or ATP (5 µM, 1 h) stimulation. LDH release was measured to assess cell membrane integrity (*n* = 5). (C) PMA-induced THP-1 cells received the same treatment as described in (A), and ELISA was used to quantify the levels of IL-1β and IL-18 in the culture supernatants (*n* = 5). (D) PMA-differentiated THP-1 cells received the same treatment as described in (A). The content of pyroptosis-associated protein was determined by Western blotting analysis (*n* = 3). Data are presented as mean ± SD. Figure S2. Meloxicam blocks the assembly of NLRP3 inflammasomes. (A) PMA-induced THP-1 cells were pretreated with meloxicam (100 µM) for 2 h prior to being stimulated with LPS (1 µg/mL, 18 h) plus Nigericin (10 µM, 1 h). Co-IP was employed to detect the interactions among NLRP3, ASC, and pro-Caspase-1. Figure S3. Meloxicam suppresses p38 phosphorylation and downregulates the protein levels of TXNIP and CBP. (A, B) PMA-differentiated THP-1 cells were pretreated using meloxicam (100 µM, 2 h) prior to incubation with LPS (1 µg/mL, 18 h) plus ATP (5 µM, 1 h). Total and phosphorylated p38, ERK, JNK, as well as TXNIP levels and CBP, were detected by Western blotting analysis (*n* = 3). Data are presented as mean ± SD. Figure S4. Meloxicam reduces intracellular ROS accumulation. (A) THP-1 cells were induced with PMA and then pretreated using meloxicam (100 µM) for 2 h, subsequently stimulated with LPS (1 µg/mL, 18 h) plus Nigericin (10 µM, 1 h) (*n* = 5). Intracellular ROS accumulation was assessed. Data are presented as mean ± SD.

## Abbreviations

The following abbreviations are used in this manuscript:

Mel	meloxicam
ROS	reactive oxygen species
CLP	cecal ligation and puncture
TXNIP	thioredoxin-interacting protein
ALI	acute lung injury
ARDS	acute respiratory distress syndrome
MODS	multiple organ dysfunction syndrome
NLRP3	nucleotide-binding oligomerization domain (NOD)-like receptor family pyrin domain-containing 3
ASC	apoptosis-associated speck-like protein containing a Caspase recruitment domain
NSAID	nonsteroidal anti-inflammatory drug
COX-2	cyclo-oxygenase-2
SN	Supernatant
H&E	hematoxylin and eosin
ELISA	enzyme-linked immunosorbent assay
ΔΨm	mitochondrial membrane potential
GSDMD	gasdermin D
GSDMD-NT	the N-terminal fragment of gasdermin D
CBP	CREB-binding protein
MAPK	mitogen-activated protein kinase
RT-PCR	reverse transcription polymerase chain reaction
Co-IP	co-immunoprecipitation
TEM	transmission electron microscope
