# Supplementary material for: Meloxicam Alleviates Sepsis-Induced Lung Injury by Inhibiting Pyroptosis Through CBP/TXNIP/p38 Signaling Pathway
Source: Pharmaceuticals (Basel). 2026 Jun 12;19(6):929. doi: 10.3390/ph19060929 (PMC13305203; doi:10.3390/ph19060929)
Supplement: Supplementary file 1 [file pharmaceuticals-19-00929-s001.zip › pharmaceuticals-4339261-supplementary.pdf]

Fig.S1

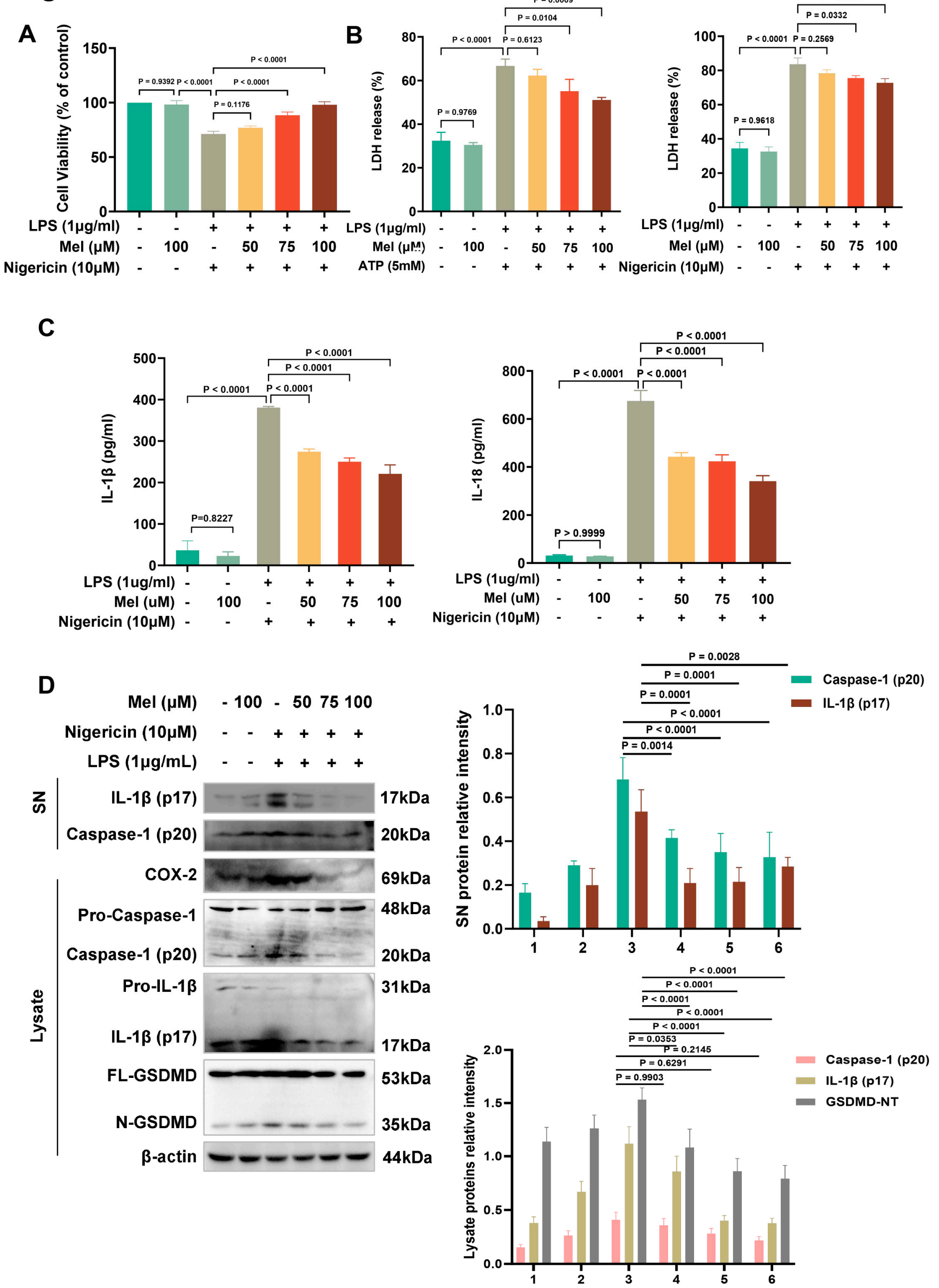

Figure S1. Meloxicam exerts a dose-dependent inhibitory effect on macrophage pyroptosis. **(A)** After being pretreated with meloxicam (50, 75, or 100 μM) for 2 hours, PMA-induced THP-1 cells were incubated with LPS (1 μg/mL, 18 hours), followed by Nigericin (10 μM, 1 hour). The CCK-8 assay was used to evaluate cell viability (n=5). **(B)** PMA-induced THP-1 cells were pretreated with meloxicam (50, 75, or 100 μM) for 2 hours, prior to LPS (1 μg/mL, 18 hour) plus Nigericin (10 μM, 1 hour) or ATP (5 μM, 1 hour) stimulation. LDH release was measured to assess cell membrane integrity (n=5). **(C)** PMA-induced THP-1 cells received the same treatment as described in (A), and ELISA was used to quantify the levels of IL-1β and IL-18 in the culture supernatants (n=5). **(D)** PMA-differentiated THP-1 cells received the same treatment as described in

(A). The content of pyroptosis-associated protein was determined by Western blotting analysis (n=3). Data are presented as mean ± SD.

Fig.S2

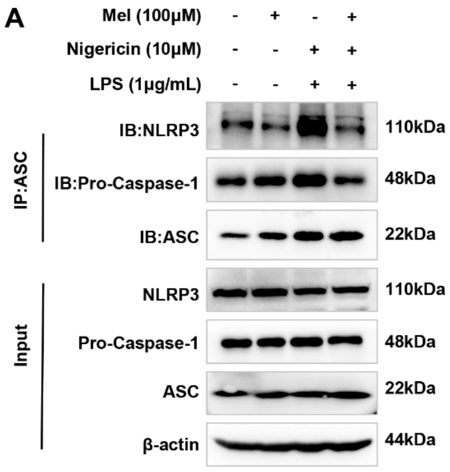

Figure S2. Meloxicam blocks the assembly of NLRP3 inflammasomes. (A) PMA-induced THP-1 cells were pretreated with meloxicam (100 μM) for 2 hours prior to being stimulated with LPS (1 μg/mL, 18 hours) plus Nigericin (10 μM, 1 hour). Co-IP was employed to detect the interactions among NLRP3, ASC, and pro-Caspase-1.

Fig.S3

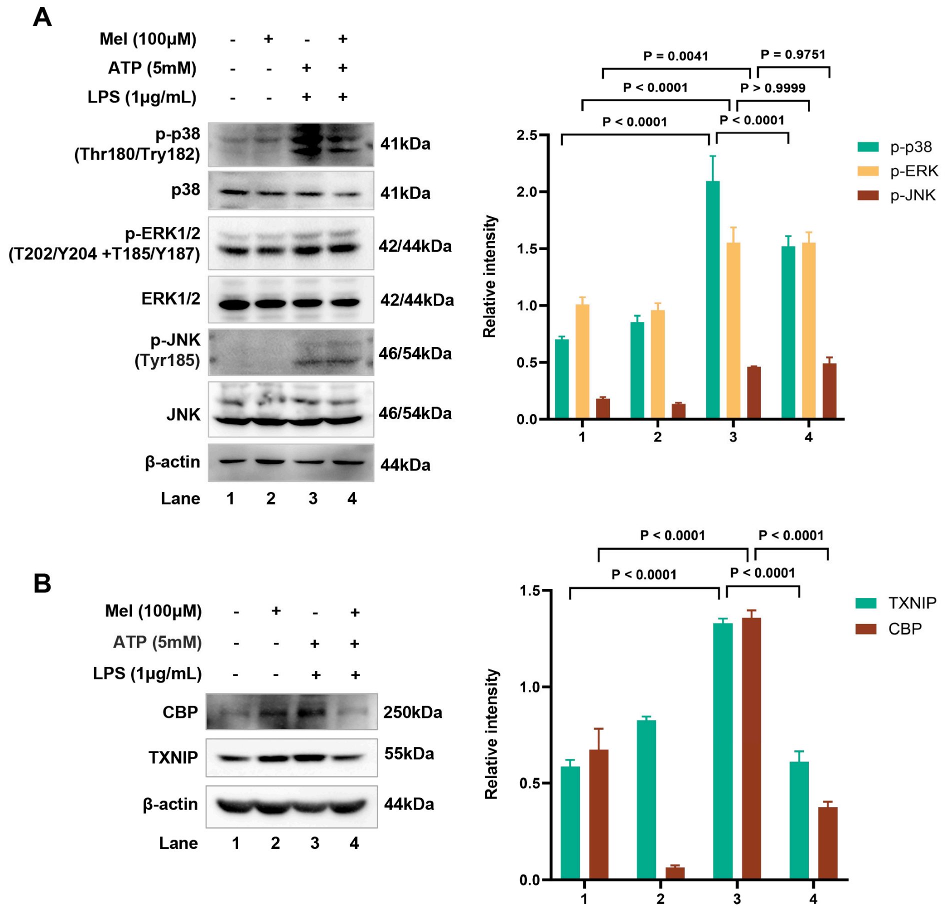

Figure S3. Meloxicam suppresses p38 phosphorylation and downregulates the protein levels of TXNIP and CBP. **(A, B)** PMA-differentiated THP-1 cells were pretreated using meloxicam (100 μM, 2 hours) prior to incubation with LPS (1 μg/mL, 18 hours) plus ATP (5 μM, 1 hour). Total and phosphorylated p38, ERK, JNK, as well as TXNIP protein levels and CBP, were detected by Western blotting analysis (n=3). Data are presented as mean ± SD.

Fig.S4

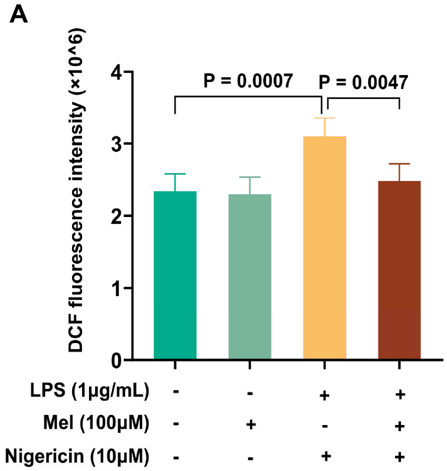

Figure S4. Meloxicam reduces intracellular ROS accumulation. (A) THP-1 cells were induced with PMA and then pretreated using meloxicam (100 μM) for 2 hours, subsequently stimulated with LPS (1 μg/mL, 18 hours) plus Nigericin (10 μM, 1 hour) (n=5). Intracellular ROS accumulation was assessed. Data are presented as mean ± SD.
